# Application of alizarin colorimetric measurements for quantification of amine extraction by model food simulants from epoxy polymer

**DOI:** 10.1186/2193-1801-2-593

**Published:** 2013-11-06

**Authors:** Tomasz Jeliński, Piotr Cysewski, Edwin Makarewicz

**Affiliations:** Department of Physical Chemistry, Collegium Medicum, Nicolaus Copernicus University, Kurpińskiego 5, 85-950 Bydgoszcz, Poland; Division of Materials Chemistry and Protective Coatings University of Technology and Life Sciences, Faculty of Chemical Technology and Engineering, Seminaryjna 3, 85-326 Bydgoszcz, Poland

**Keywords:** Epoxy resin, Alizarin, Triethylenetetramine, Food simulant, Residual amine, Curing

## Abstract

A simple and straightforward method has been proposed for quantification of residual amine in cured epoxy resin. Non-bounded triethylenetetramine was extracted from epoxy polymer and determined via spectrophotometry using alizarin chromophore. Four solvents commonly used as food simulants, namely water, 95% ethanol, 10% ethanol and 3% acetic acid were examined. Released amine induces changes in the absorption spectrum of alizarin, by decreasing the intensity of the maximum at 430 nm band and mutually increasing the 527 nm band. These changes were proportional to the amounts of amine concentration in samples. The statistical significance of obtained calibration curves was validated. Among studied solvents, the highest amine release was observed for water solution and 3% acetic acid, that is approximately 7% w/w. The maximal amount of residual amine extracted with 95% ethanol was about 1.25%, while for 10% ethanol this amount was 2%. The effect of aging of the samples and exposure to artificial sunlight were also examined. The proposed method has been proven to be fast, low cost and directly applicable for analysis of typical epoxy resins.

## Introduction

Epoxy resins are widely applied as coatings, adhesives, construction composites etc. (Muskopf and McCollister [2002]; Petrie [2006]; Wicks et al. [2007]). Common curing agents used with epoxy resins are aliphatic and aromatic polyamines (Petrie [2006]; Prolongo et al. [2003]; Wicks et al. [2007]). However, the reaction of amine groups with oxirane rings is never complete, because the conversion degree is restricted by resin vitrification and therefore, residual amine hardeners can migrate from the cured polymer. This may cause serious problems due to the known toxicity of aliphatic and aromatic amines (Hughes et al. [1995]; Patnaik [2007]; Smith [2010]). There are several examples of contact dermatitis resulting from the use of amine compounds in modified polymers (Conde-Salazar et al. [2004]; Soto et al. [1989]), and also the health problems caused by amine hardeners in epoxy systems have been studied shortly after the introduction of epoxy resins into modern industry (Bourne et al. [1959]). After many years of usage of epoxy resins in different industrial and commercial applications, the problem of health hazards caused by such systems is still not completely examined, although many published works have focused on allergies, caused by both the epoxy resin itself (Akita et al. [2001]; Kumar and Freeman [1999]; Sasseville [1998]) as well as resin modifiers, such as hardeners (Bachanek et al. [2005]; Bray [1999]). For that reason, determination of non-bounded amine hardener is an important analytical task. Chromatographic methods are the ones mostly used (Dopico-Garcia et al. [2010]; Paseiro-Cerrato et al. [2010]) however, gas chromatography has limited applicability because of low volatility of multifunctional amines. Near-infrared spectroscopy is a convenient and rapid technique offering reliable assessments of amine conversion degree (Escola et al. [2005]; Prolongo et al. [2003]). Direct spectrophotometry and spectrofluorimetry are rather less applicable to aliphatic amines, because they do not possess characteristic UV–VIS absorbance or fluorescence properties. On the other hand, both the techniques became fully applicable when appropriate colored indicator is used (Ajayakumar and Mukhopadhyay [2009]; Bao et al. [2010]; Basurto et al. [2005]; Comes et al. [2004]; García-Acosta et al. [2006]; Liu et al. [2006]; Oliveri and Di Bella [2011]; Staneva et al. [2007]). One of the promising indicators for low-polar solvents is alizarin (1,2-dihydroxy-9,10-anthraquinone) (del Río et al. [2010]). Anthraquinone derivatives in general are known as analytical reagents (Hosseini and Asadi [2009]; Mitic et al. [2007]). Alizarin itself is applied in chemical analysis as well (Chamsaz et al. [2000]; Feng et al. [2011]). Alizarin exists in three forms of different color, namely the protonated form and two deprotonated forms corresponding to the monoanion and dianion (Das et al. [2002]; Cysewski et al. [2012]; Preat et al. [2009]). The absorption maximum of the neutral non-dissociated form is located at 430 nm (Preat et al. [2009]; Say-Liang-Fat and Cornard [2011]) and the monoanionic form is characterized by an absorption maximum at around 530 nm (Quinti et al. [2003]; Savko et al. [2007]). The color change of alizarin can be modeled quite accurately based on simple protocol within TD-DFT framework, which has been shown by us in one of our earlier studies (Cysewski et al. [2012]). This makes the use of alizarin chromophore very convenient since it is sensitive to the presence of proton acceptor agents even in non-water solutions.

## Experimental

### Reagents and chemicals

Mid-viscosity epoxy resin with trade name Epidian 5 (Cedar, Poland) was used in the studies. Triethylenetetramine (TETA), known as one of the most widely used hardeners (Czub [2009]; Manthey et al. [2011]; Roskowicz and Smal [2011]; Tripathy et al. [2011]) has been purchased also from Cedar under trade name Z1. The above compounds were used without further purification. Analytical grade ethanol and acetic acid were supplied by POCH S.A. (Poland). Alizarin has been supplied by Sigma-Aldrich.

### Procedure

Stock solutions of TETA in widely accepted food simulants such as water, 95% ethanol, 10% ethanol and 3% acetic acid were prepared with the concentration of 0.160 mg/ml each. A series of diluted solutions was then prepared by adding different volumes of the solvent to a standard volume of the stock solution. Alizarin stock solutions in 95% ethanol, 10% ethanol, 3% acetic acid and methanol were prepared with the concentration of 0.4685 mmol/l. Aqueous solution was not used because the solubility of alizarin in water is very limited. Calibration samples were prepared by mixing 1.5 cm^3^ of each diluted amine solution with 1.5 cm^3^ of alizarin stock solution directly in a spectrophotometric cuvette. In such a way, three sets of calibration curves were obtained in water–methanol (50% v/v), 95% ethanol and 10% ethanol solutions. For 3% acetic acid, the alizarin stock solution was alkalized to pH equal to 12 and then the acidic solution of TETA was added as described above. Absorption spectra were recorded using a single-beam UV/VIS spectrophotometer (Merck, Spectroquant Pharo 300) with a wavelength resolution of 1 nm.

Series of epoxy polymer samples has been prepared at the hardener content equal to 8, 10, 12, 14 and 16 phr. Pre-weighted amounts of the epoxy resin and TETA were thoroughly mixed and left for 24 hours at ambient temperature for the completion of the curing process. Cured polymer pellets of about 50 mg were weighed and placed in sealed tubes filled with 3 ml of solvent for extraction. Preliminary tests showed that the extraction process is complete after 3 hours, which is documented in the Appendix. Next, 1.5 ml of the extract was taken and put into a cuvette containing 1.5 ml of the alizarin stock solution (alkalized in the case of 3% acetic acid solution). Then, the spectra were recorded in the same way as in the case of the calibration curves.

### Aging effect and sunlight exposure

In order to investigate the effect of aging of the samples on the amine release process, one series of the samples was examined immediately after preparation, while the pellets from the second series were left for six months in room temperature and then examined.

An artificial sunlight lamp was used for simulating the effect of sunlight exposure. The power density of this lamp was measured as a function of the length from the lamp to the surface containing the examined epoxy pellets. Also, the temperature was measured in order to avoid overheating of the samples. The length of 12.5 cm was chosen, giving a power density of 9.2 kW/m^2^. Since the annual solar irradiance in middle Europe (e.g. Poland) is about 1000 kWh/m^2^, a series of time intervals from 1 to 100 hours mimicked the amount of energy distributed to the samples throughout the one year span.

### Statistical analysis

Statistical analysis has been conducted as follows. Linear equations and R2 coefficients were used to determine the variance of the y-intercept (denoted as C).

The variance of the slope coefficient (denoted as B) was calculated according to the formula:1

where f is the number of degrees of freedom (for α = 0.05 the f parameter equals 3) and φ^2^ is the indetermination coefficient calculated as follows:2

where Y_i_ are the experimentally obtained values and Y_i,calc_ are the ones estimated on the basis of the obtained linear equation.

The variance of the y-intercept is connected with variance of the slope coefficient with a simple dependence:3

The standard deviation of y-intercept is then calculated as the square root of the variance:4

The limit of detection (LOD) and the limit of quantification (LOQ) where then calculated with the following equations:56

## Results and discssion

### Calibration curves

Addition of increased amounts of TETA to alizarin solution results in substantial changes of UV–VIS spectra. The absorption maximum of alizarin at 430 nm is decreased and the height of the peak at 527 nm is increased. It is obvious, that the observed spectral changes are caused by the increase of solution alkalinity and the formation of alizarin amine complex with pseudo-dissociation to anionic form. This is discussed in the Appendix section. These regularities related to color change from colorless to magenta-red are observed in all of the solvents studied.

**Figure 1:**
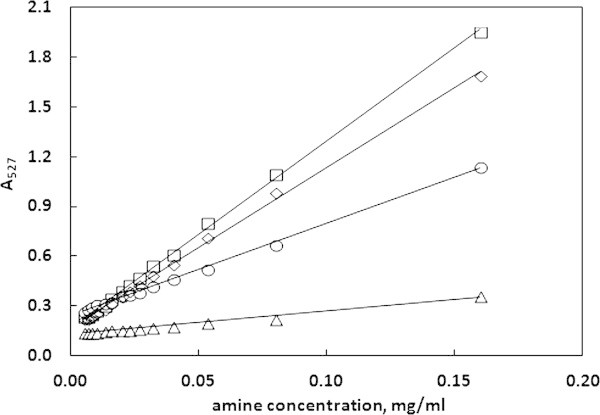
**The absorbance values at 527 nm as a function of amine concentration.** The calibration plots are obtained in water–methanol (◇), 95% ethanol (◯), 10% ethanol (△) and 3% acetic acid (☐) solutions.

The absorbance values at the wavelengths 527 nm (Figure 1) and 430 nm (Appendix) as well as the absorbance ratio (Appendix) were taken into account and tested as potential calibration curves for amine determination. The statistical analysis showed that the calibration curve corresponding to the absorbance values at 527 nm is the most reliable and was used in further analysis.

The obtained linear equations, determination coefficients, values of limits of detection (LOD) and quantification (LOQ) for this curve have been presented in Table 1, while for the two other curves in the Appendix.

**Table 1:** **Linear equations, R** ^**2**^ **coefficients, values of the standard deviation of y-intercept (SD(A)) and the values of limits of detection (LOD) and quantification (LOQ) for the calibration curve utilizing the absorbance at 527 nm**

Calibration data	95% ethanol	10% ethanol	3% acetic acid	Water
SD(C)	0.000205	0.000334	0.000621	0.000596
LOD [mg/ml]	0.000122	0.000795	0.000101	0.000203
LOQ [mg/ml]	0.000366	0.002385	0.000304	0.000610
R^2^ coefficient	0.9988	0.9922	0.999	0.998
Linear equation	y = 5.544x + 0.242	y = 1.389x + 0.128	y = 11.293x + 0.1644	y = 9.6858x + 0.165

From the data provided in Table 1 and Appendix one can see that the absorbance values at 527 nm proved to have the best linearity among the three curves. Although the curve utilizing the ratio of absorbencies has a slightly better linearity in the case of 10% ethanol and 3% acetic acid, it performs much worse in the case of 95% ethanol and water. On the other hand, the curve utilizing the absorbance values at 430 nm has a good linearity for 95% ethanol and water, although worse than the '527 nm’ curve, but it performs much worse in the case of 10% ethanol and 3% acetic acid. The above observations are also confirmed by the LOD and LOQ values for these curves. Also, the solvents used influenced both the detection and quantification limits. The best sensitivity is obtained in the case of 3% acetic acid, while the worst sensitivity characterizes 10% ethanol, although the limits of detection and quantification are satisfactory for all the solvents. One can infer, that the most appropriate calibration curve for TETA determination is provided with the absorbance at 527 nm. Besides, all four food simulant solvents may be used for analytical purpose, with conjunction of alizarin chromophore.

### Quantification of amine extraction

**Figure 2:**
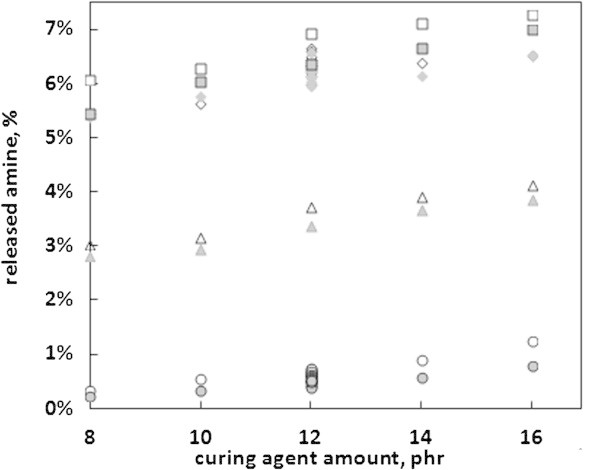
**Amounts of residual amine extracted from cured epoxy resin with water (◇), 95% ethanol (◯), 10% ethanol (△) and 3% acetic acid (☐).** The determinations performed using calibration with 527 nm absorbance for pellets after 24 hours of curing (white)and pellets after six months in room temperature (grey).

The cured epoxy resin pellets, both freshly prepared and left for six months in dark and ambient conditions, were extracted by four different solvents and the amount of amine released was determined using the calibration curve utilizing the absorbance at 527 nm. The obtained results are presented in Figure 2. Linear dependence between the residual amine amount in cured epoxy resin and the initial amine amount is observed with all the solvents applied. The polarity of the solvent affected the amount of amine extracted from the polymer. For samples examined immediately after the curing process the amount of released amine varies from 0.35% to 1.25% in the case of 95% ethanol, from 3% to 4% in the case of 10% ethanol, from 5.5% to 6.5% in the case of water solution and from 6% to 7.25% in the case of 3% acetic acid. For the samples that were left for six months the amounts of released amine were smaller. Measurements for 10 pellets containing the same amount of amine, both fresh and left for six months, were performed for water and 95% ethanol. Interestingly, statistically significant differences between the two series of pellets were noticed in the case of 95% ethanol solution, while in the case of water solution, these differences were negligible. The results obtained for pellets containing 12 phr of amine are also presented in Figure 2. In the case of 95% ethanol the standard deviations were 0.5% and 0.6% for freshly prepared pellets and those left for six months, respectively. The statistical significance was estimated based on P values, obtained using two-way heteroscedastic statistical test (*T*-test). In the case of water the standard deviations were 0.17% and 0.21%, while conducting the same test leads to the conclusion that the differences between populations are not statistically important.

The polarity of TETA results in very high affinity towards water, which was documented by extraction experiments. Having the above in mind, the lack of differences in the amounts of released amine for fresh samples and samples aging for six months suggests that there was no further progress in cross-linking reaction during this period of time. However, it is interesting to see, that such statistically significant differences occur in the case of much less polar 95% ethanol solution. This may suggest that during the aging of the sample partial penetration of the amine inside the polymer takes place. However, this physical process leads to such a small change of affinity towards water, that the extraction with the use of this polar solvent causes the release of all, or at least the majority, of the unbounded amine. Also, it can be suggested that the amine sorption process is caused by intermolecular interactions weaker than those stabilizing amine solutions in water, but stronger than those of TETA-ethanol system. Thus, residual amounts of amine that are not chemically bounded can be released to water. Also, wet surfaces coated with epoxy polymers are probably rich in extracted portions of amine, which poses some ecological challenge if such polymers are used. On the other hand, this releasing process occurs only for limited time period that is restricted to total amount of free amine in samples. Since water is a very effective extraction media it seems to be rational to propose a post-polymerization process of immersion in water as a natural way of freeing the polymer from the residual amine.

**Figure 3:**
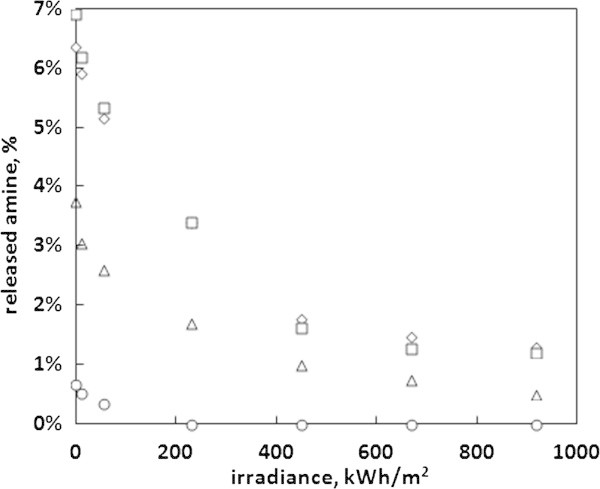
**Amounts of residual amine extracted from cured epoxy resin after exposure to artificial sunlight with water (◇), 95% ethanol (◯), 10% ethanol (△) and 3% acetic acid (☐) as the function of the irradiance of the light source.**

The results of the exposure of the cured epoxy pellets to artificial sunlight are presented in Figure 3. As it could be expected, the increased temperature and UV radiation induced a progress in the cross-linking reaction. This was documented by the decrease in the amounts of extracted residual amine. When plotting the amount of extracted amine against the irradiance of the light source, one can see its exponential decrease. In the case of water and 3% acetic acid, the amount of released amine with the maximum irradiance used was about 1.2%, which is more than 5 times less than for the samples that did not undergo light exposure. In the case of 10% ethanol solution the minimal amount reached 0.5%, while in the case of 95% ethanol the released amount of amine with the maximum irradiance could not be determined and the smallest determinable amounts of amine were extracted with 55 kWh/m^2^. This effect was accompanied by a change of color of the epoxy pellets, which gradually turned yellow. This color change shows that the ecologically valuable decrease in the amount of released amine may not come in pair with the desired mechanical or technological properties of the epoxy polymer.

## Conclusions

A new, simple and accurate method of determination of residual amine in epoxy polymer has been developed. The method utilizes the susceptibility of alizarin to undergo color changes in proton accepting media. THETA, acting as a base, induces changes in the alizarin chromophore which enables the use of spectrometric methods in quantitative determinations. The calibration plot corresponding to the absorbance at 527 nm was identified as being particularly suitable for determination of residual amine amount. The sensitivity of the developed method is fully adequate for typical epoxy resin formulations. It has been shown that the quantity of released amine is the largest in the case of water and 3% acetic acid(reaching 7%) and the smallest in the case of 95% ethanol (slightly above 1%). Such results indicate that the amounts of released amine are relatively large and therefore the release of amine from the epoxy polymer is ecologically important, since amines create significant health hazards for humans, such as dermatitis. The post-polymerization immersion of epoxy polymer in water may be used as a simple method of freeing the polymer from the residual amine. The proposed analytical method proved its usefulness in examining two different effects, namely the aging of the samples and their exposure to artificial sunlight. It was also demonstrated that the aging of the samples causes a statistically important difference in the residual amine extraction for 95% ethanol but no such difference can be observed in the case of water. This might suggest, that there is no further progress in cross-linking reaction during this time and the nature of this effect is only diffusional. Also, the exposure of the samples to artificial sunlight caused a progress in the cross-linking reaction and a resulting decrease in the amounts of extracted residual amine.

## Appendix

### Extraction time measurements

**Figure 4:**
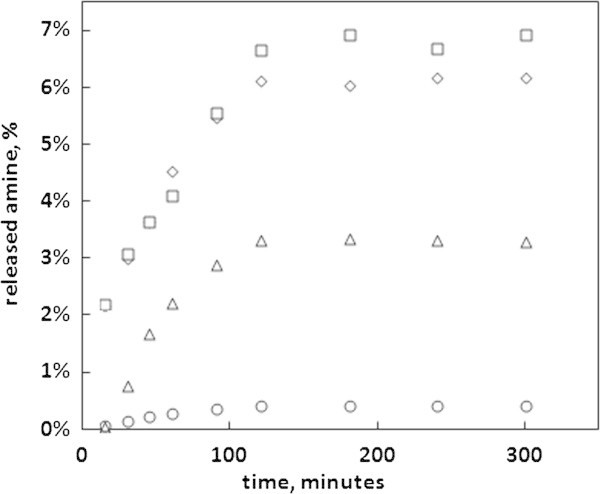
**The amine release as a function of extraction time, for water–methanol (◇), 95% ethanol (◯), 10% ethanol (△) and 3% acetic acid (☐) solutions.**

Before the quantification of amine release it was necessary to determine the time in which the extraction of cured epoxy pellets is complete. Therefore, the pellets were extracted in all the solvents for different time periods and the obtained results are shown in Figure 4. A gradual increase of the amount of extracted amine was observed during time but after 120 minutes of extraction the amount of extracted amine remained unchanged despite the increasing extraction time. Therefore, an optimal value of 3 hours has been chosen as the extraction time.

### Calibration curves less suitable for amine determination

**Figure 5:**
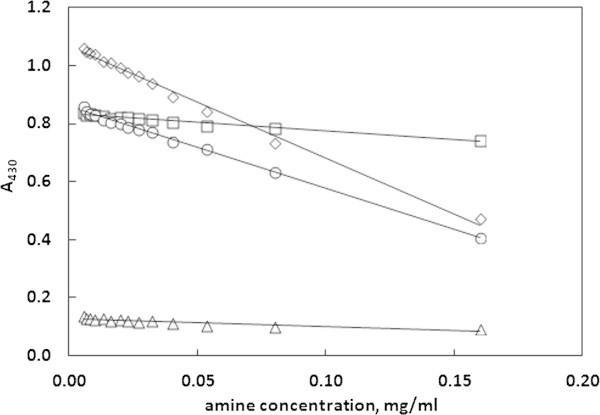
**The absorbance values at 430 nm as a function of amine concentration.** The calibration plots are obtained in water–methanol (◇), 95% ethanol (◯), 10% ethanol (△) and 3% acetic acid (☐) solutions.

**Figure 6:**
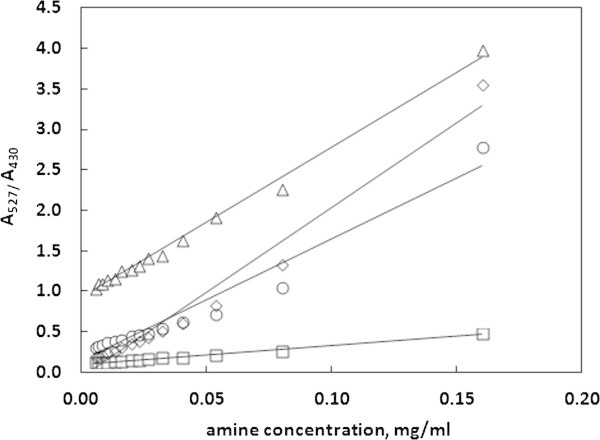
**The values of the absorbencies ratio as a function of amine concentration.** The calibration plots are obtained in water–methanol (◇), 95% ethanol (◯), 10% ethanol (△) and 3% acetic acid (☐) solutions.

The calibration curve corresponding to the absorbance at 527 nm wavelength was chosen as the most appropriate one for residual amine determination because of its good linearity in all the solvents and satisfactory values of detection and quantification limits. However, the two other considered curves also performed well in some cases. The curve utilizing absorbance values at 430 nm wave length is suitable for all the solvents except 10% ethanol, while the curve utilizing the ratio of absorbencies at 430 nm and 527 nm wavelengths is less suitable for 95% ethanol. The two considered here calibration curves are presented in Figure 5 and Figure 6. The obtained linear equations, determination coefficients, values of limits of detection (LOD) and quantification (LOQ) for these curves have been presented in Table 2 and Table 3.

**Table 2:** **Linear equations, R** ^**2**^ **coefficients, values of the standard deviation of y-intercept (SD(A)) and the values of limits of detection (LOD) and quantification (LOQ) for the calibration curve utilizing the absorbance at 430 nm**

Calibration data	95% ethanol	10% ethanol	3% acetic acid	Water
SD(C)	0.000217	0.001330	0.000484	0.000857
LOD [mg/ml]	0.000254	0.016130	0.002612	0.000733
LOQ [mg/ml]	0.000763	0.048400	0.007838	0.002201
R^2^ coefficient	0.9975	0.8532	0.9746	0.992
Linear equation	y = -2.823x + 0.860	y = -0.272x + 0.127	y = -0.612x + 0.837	y = -3.857x + 1.068

**Table 3:** **Linear equations, R** ^**2**^ **coefficients, values of the standard deviation of y-intercept (SD(A)) and the values of limits of detection (LOD) and quantification (LOQ) for the calibration curve utilizing the ratio of absorbencies**

Calibration data	95% ethanol	10% ethanol	3% acetic acid	Water
SD(C)	0.020758	0.003153	0.000619	0.019432
LOD [mg/ml]	0.004527	0.000560	0.000979	0.003051
LOQ [mg/ml]	0.013582	0.001680	0.002938	0.009154
R^2^ coefficient	0.9564	0.9945	0.9904	0.97
Linear equation	y = 15.131x + 0.131	y = 18.583x + 0.9191	y = 2.0863x + 0.1079	y = 21.016x-0.0621

### The pH values of Alizarin with TETA stock solutions

The pH value of the alizarin-TETA system is mostly dependant on the amine concentration. Since alizarin has hydroxyl groups in its structure, in water it undergoes reaction (7):7

Therefore, is not surprising that slightly acidic pH values are observed. When the amine is added reaction (8) takes place:8

**Figure 7:**
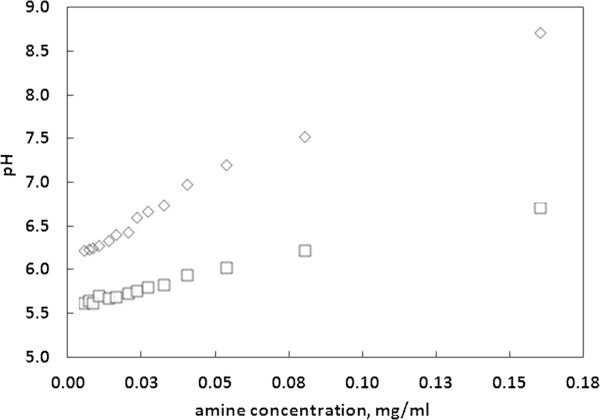
**pH values of diluted TETA stock solutions as a function of amine concentration, for water–methanol (◇) and 3% acetic acid (☐) solutions.**

This reaction is the source of hydroxide ions, the presence of which shifts the balance of reaction (7) towards AZ-O^-^, that is the deprotonated form of alizarin, responsible for the color change of the alizarin solution. This can be observed in Figure 7. Similar conclusions can be drawn for other non-acidic solutions, as methanol or acetone.

### Quantification of amine release from 3% acetic acid

In the case of 3% acetic acid, the initial solution of alizarin was alkalized to pH = 12 since alizarin is not sensitive in the range of pH characterizing acidic solutions. Still, the system is sensitive to changes of amine amount, as it was demonstrated in Figure 7. Unfortunately, artificial rise of pH unables the direct determination of amine amount based on spectroscopic response of alizarin chromophore. In this situation, the system is much more complicated what can be described by the existence of the following equilibria:9101112

These additional equilibria can be interpreted in terms of several buffers formed by acetic acid/NaOH, acetic acid/amine and with a lesser extent alizarin/amine and alizarin/NaOH. Despite a complicated interrelation between chemical species, there is a linear response of pH with respect to the added amount of TETA. The rise of amine concentration increases AC^-^ concentration and at the same time shits the balance of reactions (9) and (10) toward reactants. Consequently, a decrease of acidity is associated with promotion of reaction (12), what can be observed by spectroscopic measurements. Thus, despite a complicated equilibriums system, it can be still used for quantification of amine concentration, taking into account the relative change of absorption bands of alizarin. For this purpose the calibration curve was modified according to the following formula:

where: A_H2O_ (λ530) is the absorbance of water–methanol solution for given amine concentration; A_Ac_(λ527,C_x_) is the absorbance of the 3% acetic acid solution for the same amine concentration and A_Ac_(λ527,C_0_) is the absorbance of the 3% acetic acid modified with NaOH. This means that the relative change of absorption in the case of acetic acid solution is used instead of a direct one.
